# Methylprednisolone Protects Human Pancreatic Beta Cells From Inflammation-induced Damage

**DOI:** 10.1097/TXD.0000000000001770

**Published:** 2025-05-12

**Authors:** Miriam Paz-Barba, Iris J.A. Peters, Natascha de Graaf, Françoise Carlotti, Eelco J.P. de Koning

**Affiliations:** 1 Department of Internal Medicine, Leiden University Medical Centre, Leiden, the Netherlands.

## Abstract

**Background.:**

Methylprednisolone is a glucocorticoid often used for immunosuppressive induction therapy or treatment of rejection in the context of organ transplantation and preservation of long-term function. In pancreas and islet transplantation, there is more reluctance to use high-dose methylprednisolone when there is suspicion of rejection, partly due to its hyperglycemic effects and doubts about the rescue of islet function. Here we investigated the functional and molecular effects of high-dose methylprednisolone on human pancreatic beta cells in an inflammatory environment, focusing on the nuclear factor kappa B and endoplasmic reticulum stress pathways.

**Methods.:**

We exposed primary human islets or human beta cells to proinflammatory cytokines in the presence or absence of methylprednisolone for 3 d and characterized its effects on beta-cell death, function, gene and protein expression, and secretion of inflammatory molecules.

**Results.:**

Methylprednisolone prevented cytokine-induced beta-cell failure and death (57% decrease in caspase 3/7 activation [*P* < 0.05]) after 72 h. This protective effect was associated with an 80% attenuation of the inflammatory cytokine gene *IL-8* (80%, *P* < 0.01), the proapoptotic nuclear factor kappa B–related gene *NFKB2* (26%, *P* < 0.05), and endoplasmic reticulum stress gene *ATF3* (48%, *P* < 0.05) during cytokine treatment.

**Conclusions.:**

We propose that short-term treatment with methylprednisolone is beneficial for beta-cell health under inflammatory conditions which can be relevant in periprocedural pancreas or islet transplantation, and treatment of graft rejection.

## Graphical abstract

**Figure:**
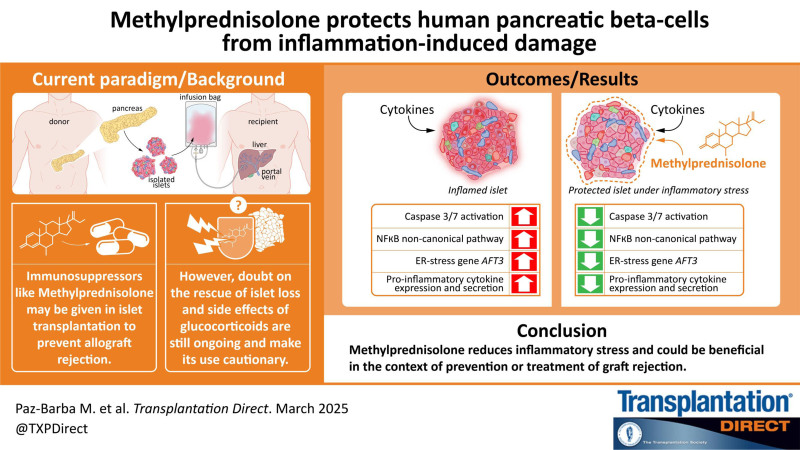

Pancreas and islet transplantation are beta-cell replacement treatments in a small group of patients with type 1 diabetes.^1,2^ It can lead to insulin independence when there is full graft function^3,4^ or, with partial graft function, can lead to stabilization of glycemic control, reduction of hemoglobin A1c,^5^ prevention of severe hypoglycemic events, and improvement of hypoglycemia awareness.^6^ Unlike pancreas transplantation, the initial loss of endocrine tissue after islet transplantation is common and is believed to happen due to a combination of factors, including instant blood-mediated inflammatory response^7^ and hypoxia.^8^

High-dose glucocorticoids (GCs) such as methylprednisolone (MP) are extensively used in solid organ transplantation due to their anti-inflammatory and immunosuppressive properties.^9-11^ Although multiple doses of MP are commonly used as part of immunosuppressive induction in pancreas transplantation, there is a reluctance to use similar high-dose GCs in islet transplantation. In both pancreas and islet transplantation, when there is suspicion of (acute cellular) allograft rejection with hyperglycemia and the necessity for insulin, there is a similar reluctance to use high-dose GCs partly due to its hyperglycemic effects^12^ and the notion that irreversible islet damage has already occurred. However, we recently showed that islet transplant recipients receiving high-dose MP therapy because of suspected allograft rejection had improved C-peptide concentrations and metabolic outcomes compared with those who had not received such immunosuppressive regime.^13^ These results indicate that MP can potentially rescue beta-cell function.

GCs like MP exert their anti-inflammatory effects mainly via inhibition of the proinflammatory nuclear factor kappa B (NF-κB) pathway,^14^ which has been extensively studied in non–islet cells. MP has a stronger binding affinity to the GC receptor compared with other steroids. After binding of MP to the GC receptor,^15^ it undergoes nuclear translocation where it performs its genomic actions and alters target gene expression, for instance by inhibiting the binding of NF-κB to DNA^16^ or increasing the expression of the inhibitory NF-κB protein IκBα.^17^ As a result, MP’s targeting of the NF-κB pathway has been shown to inhibit the expression of proinflammatory cytokines such as tumor necrosis factor-α, interleukin (IL)-1, and IL-6.^16-18^ In beta cells, the canonical and noncanonical NF-κB pathways, mediated by p65:p50 and p100/p52:relB protein complexes, respectively, are known to be activated by cytokines.^19,20^ However, it is unclear how MP affects this pathway in human islet cells under inflammatory stress during islet transplantation or islet rejection.

Here, we investigated the role of MP in human beta cells exposed to a proinflammatory environment.

## MATERIALS AND METHODS

### Cell Culture

Primary human islets were isolated from donor pancreas obtained through the Eurotransplant multiorgan donation program. Islets were used for research only if they could not be used for clinical purposes and if research consent was present, according to Dutch national laws. Islets with a purity of >75% were cultured in ultra-low attachment plates and maintained in CMRL 1066 (Corning, 99-663-CV, 1 g/l glucose) supplemented with 10 mmol/L 4-(2-hydroxyethyl)-1-piperazineethanesulfonic acid, 1.2 mg/mL nicotinamide, 10% fetal calf serum, 2 mmol/l l-glutamine, 100 units/mL penicillin, 100 μg/mL streptomycin, 20 μg/mL ciprofloxacin, and 50 μg/mL gentamycin. Donor characteristics are listed in **Tables S1 and S2** (**SDC**, http://links.lww.com/TXD/A743).

The human EndoC-βH1 beta-cell line^21^ was obtained from Univercell Biosolutions (Toulouse, France). The cells were seeded in Matrigel/ECM (extracellular matrix) coated plates, cultured in Dulbecco’s modified essential medium (Invitrogen, 1 g/l glucose), supplemented with 10 mmol/L nicotinamide, 5.5 g/mL transferrin, 6.7 ng/mL selenite, 100 units/mL penicillin, 100 μg/mL streptomycin, and 50 μM β-mercaptoethanol.

### MP Treatment

Human islets and EndoC-βH1 cells were exposed for 72 h to a proinflammatory cytokine mix consisting of 1 ng/mL IL-1β (401-ML, R&D systems) + 50 ng/mL interferon-γ (IFN-γ; 285-IF, R&D systems). In addition, the cells were treated with or without 2.5 µM of MP (M0639, Sigma, USA).

The concentration of MP used in this study is based on (1) the concentration of MP in blood 10 h after a high dose (1000 mg) of MP,^22^ (2) a previously used dose of MP 2 µM showing a reduction of inflammation in vitro,^23^ and (3) in vitro dose-response results performed showing inhibition of IL-8 expression with a dose of 2.5 and 25 µM but a reduction in the stimulation index with a dose of 25 µM (data not shown). Based on these considerations, a dose of 2.5 µM MP was used.

### Static Glucose-stimulated Insulin Secretion

Islet function was assessed by glucose-stimulated insulin secretion.^24^ In brief, human islets were handpicked and transferred into 96 transwell plates (MANMN4010, Merck Millipore, USA) and preincubated with KRBH (Krebs-Ringer bicarbonate 4-(2-hydroxyethyl)-1-piperazineethanesulfonic acid) buffer supplemented with 2 mM glucose (KRBH 2 mM) for 90 min, followed by sequential 1-h incubations with KRBH 2 mM and KRBH 20 mM of glucose. Supernatants were collected, and human insulin levels were measured by ELISA (10-1113-10, Mercodia, Sweden) according to the manufacturer’s instructions. At the end of the assay, the cells were lysed with lysis buffer, and DNA content was determined by Quant-iT Picogreen dsDNA assay (P7589, Thermo Fisher, USA).

### Apoptosis

For assessment of apoptosis, human islets were fixed with 4% formalin, embedded in agar, and later processed for paraffin embedding. Slices of 4 µm were cut using a microtome (RM2255, Leica, Germany), and slides were deparaffinized and subsequently rehydrated in decreasing ethanol concentrations. Antigen retrieval was performed by heating slides in 10 mM citrate buffer at pH 6.0 using a pressure cooker. Apoptotic nuclei were quantified with the in situ cell death TdT-mediated dUTP-X nick end labeling detection kit (ref 11684795910, Roche, Switzerland), following the manufacturer’s instructions. Slides were costained for insulin with C-peptide (ref GN-ID4-s, DSHB, USA) and Hoechst 33258 (H3569, Thermo Fisher). Imaging was performed with a confocal Leica SP8 WLL microscope (Leica, Germany) or Zeiss LSM 900 Airyscan (Zeiss, Germany) and quantified by using Fiji software.^25^

### Caspase 3/7 Activity

Human islets were handpicked and transferred into 96-well plates, and caspase 3/7 activity was performed following the manufacturer’s instructions (Caspase-Glo 3/7 Assay System, Promega, USA).

### Gene Expression

Gene expression was performed by quantitative reverse transcription polymerase chain reaction. RNA (250–500 ng) was obtained according to the manufacturer’s instructions (micro RNeasy kit from Qiagen, Hilden, Germany). In brief, human islets and EndoC-βH1 cells were lysed with RNA lysis buffer (Qiagen) and β-mercaptoethanol (Sigma), and isolated RNA was retro-transcribed with Moloney Murine Leukemia Virus reverse transcriptase (Invitrogen, Waltham, MA), oligo(dT)s (Qiagen), Deoxynucleotide triphosphate (dNTP) from Promega, USA, dithiothreitol (DTT) (Invitrogen) and RNAse-OUT (ThermoFisher, USA). IQ SYBR green (170-8887, Biorad) was used for measuring gene expression, and amplification and detection were analyzed with CFX systems (Biorad, Hercules, USA). Gene expression was normalized to housekeeping genes *ACTIN* and *GAPDH,* and the 2^—^^ΔΔ^^C^_t_ method was used to calculate fold changes compared with the controls. Primers are included in **Table S3** (**SDC**, http://links.lww.com/TXD/A743).

### Western Blot

Human islets (~3000 IEQ) or EndoC-βH1 (~500 000 cells) were lysed with RNA lysis buffer + 100X protease inhibitor cocktail (8220-70737, ThermoFisher) for 10 min on ice. The protein content of the supernatant was determined with the bicinchoninic acid protein assay kit according to the manufacturer’s instructions (23225, ThermoFisher), and 10–20 μg of protein was loaded in 12% mini-PROTEAN (Tris-Glycine eXtended) (TGX) gels (561043, Biorad, USA). Blots were transferred to 0.2 μmol/L polyvinylidene fluoride membranes by using the Trans-blot turbo mini 0.2 μmol/L polyvinylidene fluoride transfer packs (170415, Biorad, USA) and were blocked with 5% Milk in phosphate buffered saline (PBS)-Tween for 1 h. Primary antibodies were incubated overnight at 4 ºC, washed 3 times with PBS-Tween buffer, and incubated with secondary antibodies for 1 h at room temperature. Blots were developed with the Supersignal West Pico PLUS Chemiluminescent substrate (Thermofisher), visualized with Biorad ChemiDoc Touch (Biorad, USA), and analyzed with ImageLab. The antibodies used are listed in **Table S4** (**SDC**, http://links.lww.com/TXD/A743).

### Luminex Cytokine Assays

Supernatant from human islets (~ handpicked 200 IEQ) was procured and analyzed for secreted cytokines and chemokines according to the manufacturer’s instructions (Bio-Plex Pro Human Cytokine 27-plex Assay, Biorad, USA).

### Statistical Analysis

Data are expressed as mean ± SEM and analyzed with GraphPad Prism (GraphPad Prism Software Inc, San Diego, CA). A paired student *t* test was used as a statistical method and statistical significance was determined as a *P* value of <0.05.

## RESULTS

### MP Prevents Inflammation-induced Beta-cell Dysfunction and Beta-cell Death

**FIGURE 1.**
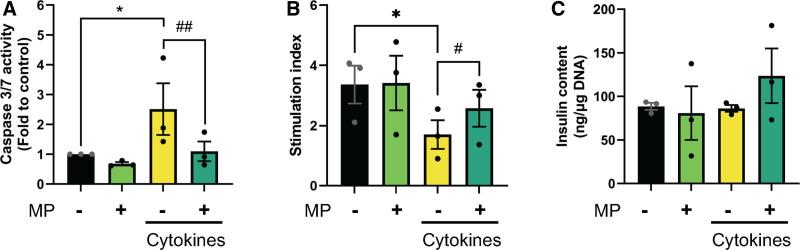
MP protects human islets from cytokine-mediated cell death. Human islets were treated with IL-1β + IFN-γ (cytokines) for 72 h, with or without 2.5 µM of MP. A, Cell death was measured by caspase 3/7 activity. B, Beta-cell function was measured in islets by static GSIS and presented as stimulation index (insulin secreted in response to 20 mM glucose relative to 2 mM glucose). C, Intracellular insulin content was measured after GSIS. Results are the means ± SEM of 3 independent experiments; *P < 0.05 compared with control and ^#^*P* < 0.05, ^##^*P* < 0.01 compared with cytokines. GSIS, glucose-stimulated insulin secretion; IFN, interferon; IL, interleukin; MP, methylprednisolone.

To investigate whether MP affects cytokine-induced beta-cell death, we exposed primary human islets to 1 ng/mL IL-1β + 50 ng/mL IFN-γ in the absence or presence of MP (2.5 µM) for 72 h. Proinflammatory cytokine treatment resulted in increased beta-cell death, as shown by enhanced caspase 3/7 activity (Figure 1A), whereas cotreatment with MP prevented this effect. A similar trend was observed by the TdT-mediated dUTP-X nick end labeling assay (**Figure S1A and B**, **SDC**, http://links.lww.com/TXD/A743).

We assessed beta-cell function in the same experimental setup. The stimulation index was reduced by 49% ± 6.7 on cytokine treatment while MP attenuated this reduction (Figure 1B). Total insulin content was not significantly affected in any of the conditions (Figure 1C). Collectively, these data indicate that MP can prevent beta-cell death and dysfunction induced by inflammation.

### MP Treatment Protects Beta Cells by Inhibiting the Noncanonical NF-κB Pathway

We hypothesized that the mechanism of action of MP involved the NF-κB signaling pathway in human beta cells. We evaluated both the canonical and noncanonical pathways.

**FIGURE 2.**
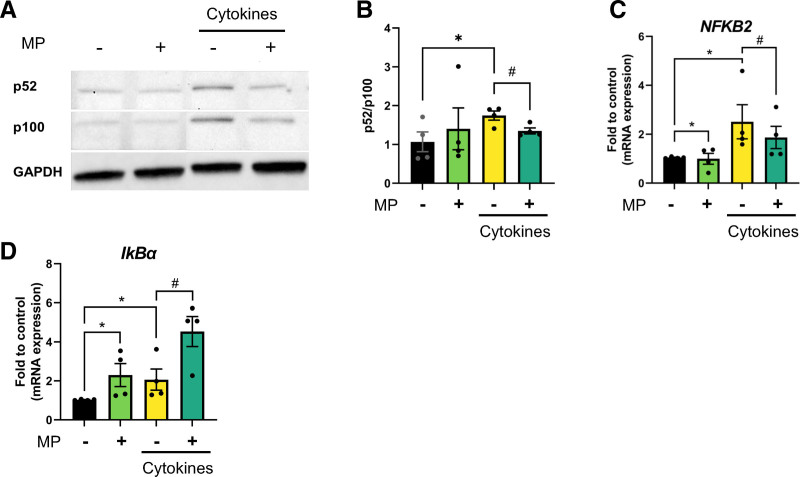
MP decreases the NF-κB signaling pathway at different levels. EndoC-βH1 cells were treated with IL-1β + IFN-γ (cytokines) for 72 h, with or without 2.5 µM of MP. A, Western blot showing protein levels of p52, p100, and GAPDH (as loading control). B, Levels of active p52 relative to inactive and unprocessed p100 are shown as p52/p100 based on quantification from the Western blot shown in (A). C, Gene expression of *NFKB2* analyzed by qPCR, normalized to control. Results are the means ± SEM of 4 independent experiments; *P < 0.05 compared with control and ^#^*P* < 0.05 compared with cytokines. IFN, interferon; IL, interleukin; MP, methylprednisolone; NF-κB, nuclear factor kappa B; qPCR, quantitative polymerase chain reaction.

Cytokine treatment activated the noncanonical NF-κB pathway, as seen by an increased p52/p100 ratio (Figure 2A and B). Interestingly, cotreatment with MP significantly prevented this effect. We also observed that MP blocked the cytokine-induced increase of *NFKB2* (p100/p52) gene expression (Figure 2C).

We found that the canonical pathway, expressed as p-p65/p65 ratio at the protein level (**Figure S2A and B**, **SDC**, http://links.lww.com/TXD/A743) and gene expression of *NFKB1* and *p65* (**Figure S2C**, **SDC**, http://links.lww.com/TXD/A743), was not affected by our cytokine treatment in EndoC-βH1 cells.

However, the NF-κB pathway inhibitor protein, IκBα, was increased on inflammation and with MP alone. In addition, we observed a synergistic effect of MP and cytokines in increasing *NFKBIA* (IκBα) gene expression (Figure 2D). The activation of IκBα, shown as increased phosphorylated IκBα (increased p-IκBα/IκBα ratio), was upregulated on inflammation and MP alone. The addition of MP to cytokines showed a tendency to further increase the activation of IκBα (**Figure S2D and E**, **SDC**, http://links.lww.com/TXD/A743).

Together, these results indicate that MP regulates the NF-κB pathway at both gene and protein levels, mainly via the noncanonical pathway.

### MP Prevents Cytokine-induced Increase in Proinflammatory Cytokines in Human Islets and Beta Cells

**FIGURE 3.**
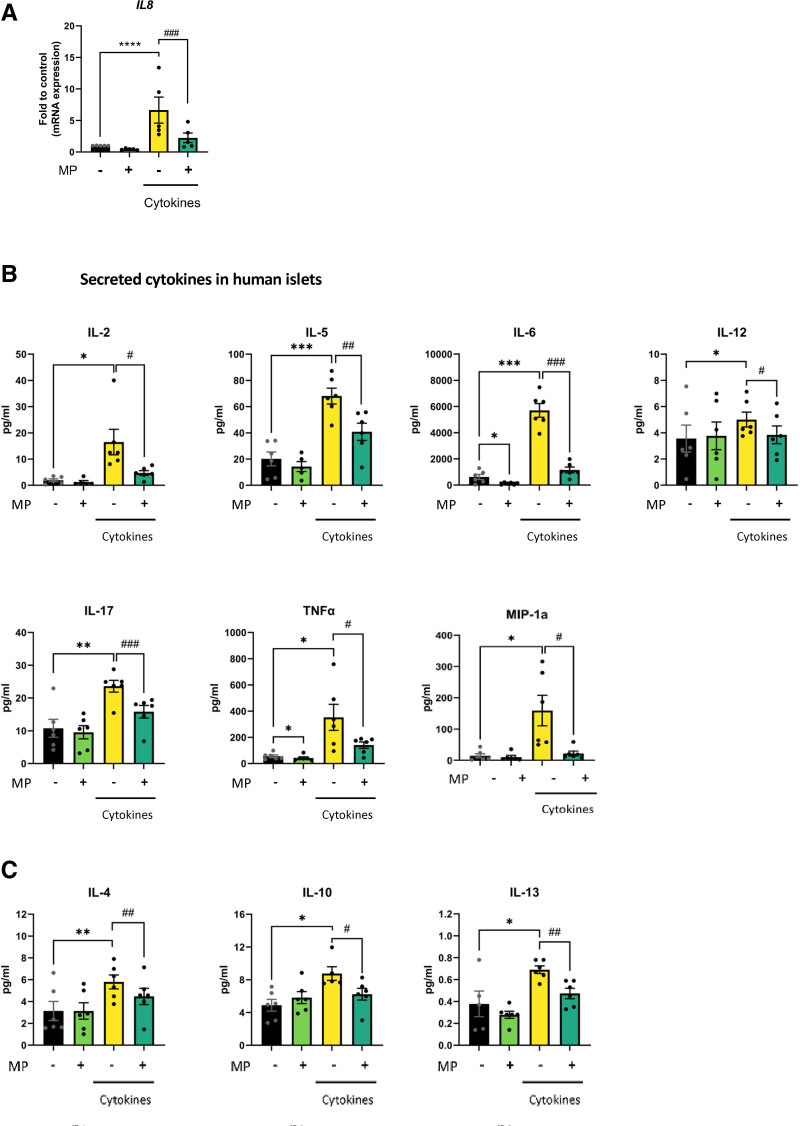
MP decreases inflammation. Human islets exposed to IL-1β + IFN-γ (cytokines) for 72 h, with or without 2.5 µM of MP. A, Gene expression of *IL8* was analyzed by qPCR and normalized to control condition. B, Multiplex immunoassay showing secreted levels of proinflammatory cytokines and chemokines analyzed by Luminex. C, Secreted levels of anti-inflammatory cytokines. Results are the means ± SEM of 5–6 independent experiments; *P < 0.05, **P < 0.01, ***P < 0.001 compared with control and ^#^*P* < 0.05, ^##^*P* < 0.01, ^###^*P* < 0.001 compared with cytokines. IFN, interferon; IL, interleukin; MP, methylprednisolone; NF-κB, nuclear factor kappa B; qPCR, quantitative polymerase chain reaction.

To further move into the molecular mechanisms underlying MP action, we assessed whether MP altered the expression of NF-κB-regulated chemokines and cytokines that are key to the inflammatory response in islet transplantation.^26,27^ IL-1β + IFN-γ upregulated gene expression of *IL8* in human islets (Figure 3A), which was attenuated by MP. Furthermore, we found that on inflammatory stress, MP inhibited the increased secretion of the vast majority of proinflammatory proteins (Figure 3B). In addition, MP also decreased the anti-inflammatory cytokines IL-4, IL-10, and IL-13 (Figure 3C). Together, these data show that MP markedly blocks inflammation in human islets exposed to proinflammatory cytokines.

### MP Inhibits Cytokine-induced Increase in ER Stress

**FIGURE 4.**
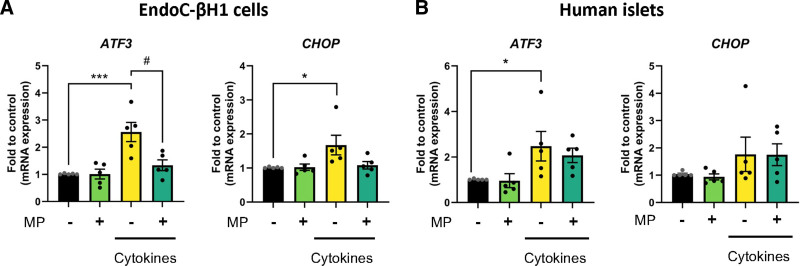
MP decreases ER stress in beta cells. EndoC-βH1 cells and human islets were treated with IL-1β + IFN-γ (cytokines) for 72 h, with or without 2.5 µM of MP. A, Gene expression of *ATF3* and *CHOP* was analyzed by qPCR and normalized to the control condition in EndoC-βH1 cells. B, Gene expression of *ATF3* and *CHOP* analyzed by qPCR and normalized to control conditions in human islets. Results are the means ± SEM of 5 independent experiments; *P < 0.05, ***P < 0.001 compared with control, ^#^*P* < 0.05, compared with cytokines. IFN, interferon; IL, interleukin; MP, methylprednisolone; qPCR, quantitative polymerase chain reaction.

ER stress has been shown to play a role in cytokine-induced beta-cell death.^28^ Treatment of EndoC-βH1 with IL-1β + IFN-γ significantly upregulated the expression of proapoptotic ER stress markers *ATF3* and *CHOP* (Figure 4A). MP blocked the inflammation-induced upregulation of *ATF3.* In primary human islets treated with cytokines, no mitigating effect of MP was observed on *ATF3* and *CHOP* gene expression (Figure 4B). Thus, although data for primary human islets are less clear, MP may also inhibit cytokine-induced ER stress, which may partly explain the reduction in cell death observed.

## DISCUSSION

There is an ongoing discussion about the use of high-dose MP after pancreas or islet transplantation to rescue beta-cell function in case islet rejection is suspected on the basis of (progressive) hyperglycemia and the need for insulin therapy. We recently showed that MP has a beneficial effect on islet graft function in islet recipients with a suspected rejection episode. However, how MP exerts its effects on human islets in this pancreas transplantation or islet transplantation context is not known. Here, we show that MP is efficient in mitigating the detrimental impact of inflammation on human islet cells.

The increased apoptosis of human islet cells and decreased beta-cell function when islets were exposed to inflammatory stress were mitigated by MP. Previous literature has shown that similar MP doses (2–2.6 µM) and other GCs either decrease insulin secretion^29,30^ or do not affect insulin secretion.^31^ In contrast, Lund et al^23^ showed that while 2 µM of MP initially reduced insulin secretion in human islets, the secretory capacity was restored and improved after 7 d. However, these in vitro studies did not account for the inflammatory environment that islets are exposed to in a transplantation or rejection setting.

We then explored the mechanism underlying the effects of MP. Although typically both the canonical and noncanonical NF-κB pathways are activated on inflammatory stimuli and crosstalk between each other, the canonical (p65:p50 protein complex) pathway is triggered in minutes while the noncanonical (p100/p52:relB protein complex) is activated in hours, leading to the expression of late-response genes.^20,32^ This could explain why, in our study, 3-d exposure to cytokines did not increase the canonical NF-κB pathway. As the role of MP in modulating the noncanonical NF-κB pathway had not been thoroughly studied before, we now show that MP significantly prevented the activation of this pathway on inflammatory triggers. Previous research focusing on the NF-κB pathway showed that the GC dexamethasone inhibited NF-κB binding activity and increased IκBα mRNA levels in human immortalized^33^ and murine immune cells.^14^ In our study, we observed that MP significantly increased IκBα gene expression in the absence or presence of cytokines.

Our results show that MP significantly prevented the increase in cytokines and chemokines from islets under inflammatory stress, which is known to occur during transplantation and rejection.^26,27^ This is in line with previous studies showing that MP decreases the production and release of proinflammatory cytokines in different cell types that are not under additional cytokine stress.^23,30,34,35^ However, we also found there was an inhibition of the anti-inflammatory cytokines IL-4, IL-10, and IL-13. Thus, while MP suppressed the expression of relevant proinflammatory cytokines and had beneficial effects on beta-cell death and function, the beneficial effects may be attenuated by a reduction in anti-inflammatory cytokines, although this would need further investigation.

Another well-studied cause of inflammation-regulated cell death in beta cells is ER stress.^28^ We observed that MP decreased the cytokine-induced expression of proapoptotic *ATF3* and *CHOP* ER stress genes. These findings are reminiscent of a study in intestinal cells showing that dexamethasone alleviated ER stress by promoting the correct protein folding and UPR gene activation.^36^ In contrast, Linssen et al^37^ reported an increase in ER stress markers after 20 h of prednisolone treatment in INS1E cells. The differences in timing, dose, GC of choice, and cell type could account for the contrasting results. In primary human islets, we were not able to validate the effect of MP on ER stress observed in human beta-cell lines. This may be explained by the presence of non–beta islet cells that could attenuate this effect, as MP has been shown to have differential transcriptional effects on immunomodulatory genes depending on the cell type studied.^38^

Our study has several limitations. Although islet inflammation is present in ischemia/reperfusion injury on pancreas transplantation, in the immediate blood-mediated inflammatory reaction in islet transplantation, and in rejection in the context of both transplantation options, our in vitro model of human isolated islet exposure to IL-1β and IFN-γ does not recapitulate the variable stressors to islets that are present in these different in vivo inflammatory situations. We only investigated MP in a dose that is relevant in the setting of peritransplant immunosuppressive induction or acute rejection therapy when high doses of MP are administered, and islets are highly stressed. The study results cannot be translated to a setting of maintenance immunosuppressive therapy with low-dose steroids and no acute stress on islets. Our studies do not answer the question of whether the full 72 h of MP is necessary for its effects. In addition, while we explored the role of MP in human islets, we did not perform experiments in a transplantation model. In another study, mice with diabetes transplanted with MP-cultured islets showed enhanced glycemic outcomes,^23^ strengthening our observations.

In conclusion, MP showed beneficial effects for human islet survival and function in an inflammatory environment. These results lend further support to the beneficial role of MP in the context of induction immunosuppressive therapy in pancreas and islet transplantation and should spur further investigations into the rescue of beta-cell function during pancreas or islet rejection, even when hyperglycemia and the need for insulin is present. Even a partial rescue of endocrine function has beneficial effects on glycemic control and patients’ quality of life.

## ACKNOWLEDGMENTS

The authors thank Els van der Beelen for the help with the Luminex experiments. They also thank Saskia du Pré (LUMC, Department of Internal Medicine) for careful reading of the article and Manon Zuurmond (LUMC) for the graphical abstract illustration.

## Supplementary Material


