# Supplementary material for: Methylprednisolone Protects Human Pancreatic Beta Cells From Inflammation-induced Damage
Source: Transplant Direct. 2025 May 12;11(6):e1770. doi: 10.1097/TXD.0000000000001770 (PMC12073852; doi:10.1097/TXD.0000000000001770)
Supplement: Supplementary file 1 [file txd-11-e1770-s001.pdf]

Supplementary Data

Table S1: *Human Islet donor information*

Donor	Islet ID	Sex	Age	BMI	Donor cause of death	Purity	Islet index
1	R213	M	66	26	Donation after circulatory death	95%	0,754
2	R223	M	61	26	Donation after circulatory death	92.5-95%	0,730
3	R205	M	19	29	Donation after circulatory death	85%	0,849
4	R197	M	73	25	Donation after circulatory death	95%	0,304
5	R199	M	29	29	Donation after circulatory death	75-93%	0,813
6	R209	F	55	24	Donation after circulatory death	75%	0,480
7	P659	F	28	25	Donation after circulatory death	85%	0,317
8	P692	M	64	33	Donation after circulatory death	80%	1,992
9	P699	M	52	25	Donation after brain death	95%	0,612
10	P664	M	29	28	Donation after circulatory death	95%	3,415

11	P713	F	64	38	Donation after circulatory death	99%	1,414
12	P714	F	58	33	Donation after brain death	98%	2,947
13	R203	F	65	21	Donation after circulatory death	95%	1,746
14	R235	F	56	30	Donation after circulatory death	98%	3,002
15	R236	M	66	25	Donation after circulatory death	75%	2,956

Table S2: *Characteristics of islet preparations after isolation before treatments*

	Purity (Mean % ± SEM, n=15)	Islet index (Mean ± SEM n=15)	IEQ decrease isolation day vs start of experiments (Mean % ± SEM, n=15)	TUNEL$^+$ beta-cells (Mean % ± SEM, n=3)*
Characteristics of the islet preparations before treatments	89.9 ± 2.2	1.4 ± 0.3	18.4 ± 7.9	10.6 ± 4.5*

*This TUNEL assay quantification corresponds to the untreated controls performed in 3 islet preparations after treatments (with/without cytokines and/or methylprednisolone).

Table S3: *List of human primers*

Gene name	5'-> 3'	3'-> 5'
GAPDH	GGAAGCTTGTCATCAATGG	TGATGATCTTGAGGCTGTTG
ACTB	TGCGTGACATTAAGGAGAAG	TGAAGGTAGTTTCGTGGATG
NFKB2	AGAGGCTTCCGATTTCGATATGG	GGATAGGTCTTTCGGCCCTTC
IL8	TCTGGCAACCCTAGTCTGCT	AAACCAAGGCACAGTGGAAC
ATF3	GTGCCGAAACAAGAAGAAGG	TCTGAGCCTTCAGTTCAGCA
CHOP	GACCTGCAAGAGGTCCTGTC	CTCCTCCTCAGTCAGCCAAG
NFKB1	GAAGCACGAATGACAGAGGC	GCTTGGCGGATTAGCTCTTTT
p65	GTGGGGACTACGACCTGAATG	GGGGCACGATTGTCAAAGATG
IKBA	GTGCTGATGTCAATGCTCAG	GGGAGAATAGCCCTGGTAGG

Table S4: *List of Antibodies*

Name	Supplier	Reference	Origin	Dilution
p52/p100	Cell signalling	4882	Rabbit	1:300
Total p65	Cell signalling	#8242	Rabbit	1:500
Phospho-p65 (Ser536)	Cell signalling	#3033	Rabbit	1:1.000
Total IκBα	Cell signalling	#4814	Mouse	1:1.000
Phospho-IκBα (Ser32/36)	Cell signalling	#9246	Mouse	1:1.000
GAPDH D16H11 XP	Cell signalling	#5174	Rabbit	1:1.000
Anti-mouse IgG HRP	Dako Cytomation	P0447	Goat	1:10.000
Anti-rabbit IgG HRP	Dako Cytomation	P0448	Goat	1:10.000

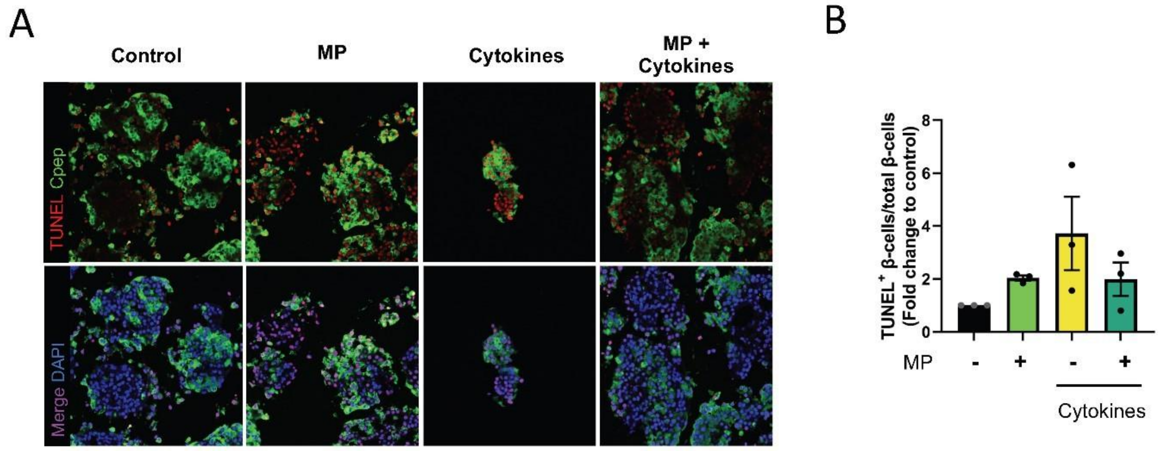

Figure S1: Effect of methylprednisolone on cytokine-induced cell death and insulin secretion. Human islets were treated with IL-1β + IFNγ (Cytokines) for 72h, with or without 2.5 µM of methylprednisolone (MP). (a) Representative immunofluorescence images of islets stained for TUNEL cell death staining (red), C-peptide (green) and nuclei (blue). (b) Percentage of TUNEL positive beta-cells compared to all beta-cells (C-peptide positive cells) in islets. Results are the means ± SEM of 3 independent experiments.

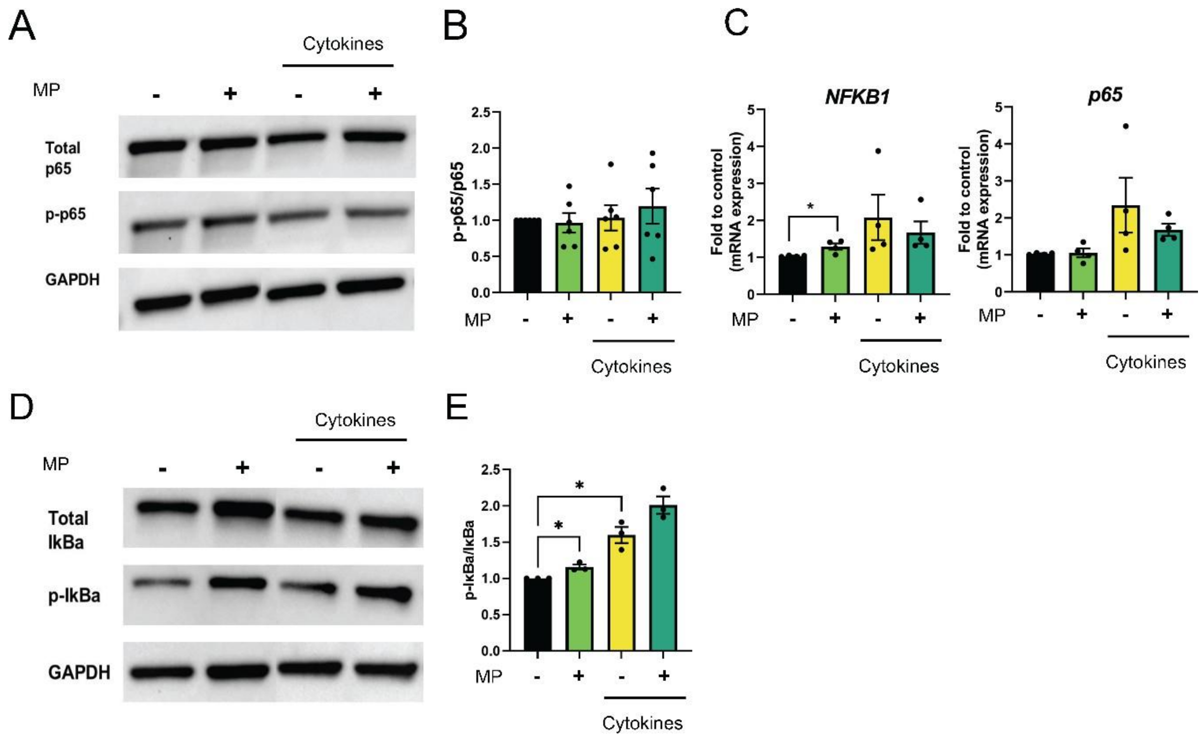

Figure S2: Methylprednisolone's effect on the canonical NFκB signaling pathway. EndoC-βH1 cells were treated with IL-1β + IFNγ (Cytokines) for 72h, with or without 2.5 µM of methylprednisolone (MP). (a) Western blot showing protein levels of total p65, phosphorylated-p65 (p-p65) and GAPDH (as loading control). (b) Levels of phosphorylated p65 (p-p65) relative to total p65 (p65) are shown as p-p65/p65 based on quantification from the Western blot shown in (a). (c) Gene expression of *NFKB1* and *p65* analyzed by qPCR, normalized to the control condition. (d) Western blot showing protein levels of total IκBα, phosphorylated-IκBα (p-IκBα) and GAPDH (as loading control). (e) Levels of phosphorylated IκBα (p-IκBα) relative to total IκBα (IκBα) shown as p-IκBα /IκBα based on quantification from Western blot shown in (d). Results are the means ± SEM of 3-6 independent experiments; *$p<0.05$ compared to Control.
